# First Histological Study of the Gastrointestinal Tract and Associated Lymphoid Structures of a Harbour Porpoise (*Phocoena phocoena*)

**DOI:** 10.3390/ani15223277

**Published:** 2025-11-13

**Authors:** Diego Pérez-Maroto, Ana Balseiro, Patricia Barroso, Ignacio Molpeceres-Diego, Antonio Fernández, Juan Francisco García Marín, Natalia García-Álvarez

**Affiliations:** 1 Animal Health Department, University of León, 24071 León, Spain; dperem10@estudiantes.unileon.es (D.P.-M.); pbars@unileon.es (P.B.); jfgarm@unileon.es (J.F.G.M.); ngara@unileon.es (N.G.-Á.); 2 Atlantic Center for Cetacean Research (CAIC), University Institute for Animal Health and Food Safety (IUSA), University of Las Palmas de Gran Canaria (ULPGC), 35413 Las Palmas, Spain; ignacio.molpeceres101@alu.ulpgc.es (I.M.-D.); antonio.fernandez@ulpgc.es (A.F.)

**Keywords:** cetacean, harbour porpoise, immunohistochemistry, gastrointestinal tract, lymph node, GALT

## Abstract

The current knowledge on the histological structure of the gastrointestinal tract (GIT) in cetaceans is based on general descriptions. The aim of this study was to characterize the histology and expression of immune cell markers in samples from the GIT and lymph nodes (LNs) in a harbour porpoise (*Phocoena phocoena*) bycaught in the Cantabrian Sea. The thickness of the histological layers of the GIT was measured, being greater in the stomach and anal canal, although no significant differences were found among any intestinal segment (*p* = 0.448). Variation in thickness, morphology of the folds, and the presence of Peyer’s patches allowed the duodenal ampulla and the distal segments to be distinguished from the rest of the intestine. An immunohistochemical technique was performed to identify the following markers: IBA1 for macrophages, CD3 for T lymphocytes, and CD20 for B lymphocytes. The distribution of immune cells varied significantly along the GIT, with higher percentages of all three cell types in the distal intestine and the anal tonsil. Within the LNs, B lymphocytes represented the predominant cell population. This study provides the first description of the histological structure of the GIT and associated lymphoid tissue in a harbour porpoise, which will be useful for future research studies.

## Simple Summary

Here, we characterized the histological structure and distribution of immune cells (macrophages and T and B lymphocytes) in the gastrointestinal tract (GIT) and associated lymphoid tissue, including lymph nodes, of a harbour porpoise (*Phocoena phocoena*) that died due to *bycatch* in the Bay of Biscay. Despite the lack of a clear distinction between the small and large intestine, the difference in thickness, folds, and the presence of Peyer’s patches allowed differentiation of the duodenal ampulla and the distal segments from the rest of the intestine. Within the lymph nodes, B lymphocytes represented the predominant cell population.

## 1. Introduction

*Cetacea* represents one of the most diverse orders of mammals, with two distinct suborders: Mysticeti, which possess baleen plates in the oral cavity, and Odontoceti, which have teeth instead [1].

The anatomy and histology of the gastrointestinal tract (GIT) of cetaceans can be found in various publications [1,2], many of them on captive animals, such as the bottlenose dolphin (*Tursiops truncatus*) [3]; however, those are general descriptions in which numerous aspects remain unclear, especially considering the wide diversity of cetacean species. In addition, the study of the normal morphology of the GIT and the cell populations of associated lymphoid tissue in wild cetaceans faces significant methodological limitations, i.e., although stranded individuals provide valuable data, they often present diseases or advanced deterioration, which can significantly alter the morphology of the GIT, compromising the representativeness of the findings. In contrast, individuals obtained through *bycatch* could be considered a suitable source for the histological characterization of the GIT, since the ultimate cause of death is attributed to fishery interaction rather than to any underlying pathological condition.

Odontocetes exhibit anatomical peculiarities in their GIT. First, these animals possess a gastric complex consisting of three distinct chambers [4]. The first compartment, the forestomach, continues the esophagus and performs a mechanical digestion function. Histologically, it is characterized by a stratified keratinized lining epithelium, reflecting its adaptation to mechanical digestion, as odontocetes do not chew their food [5]. The main stomach follows the forestomach and serves as the principal chamber for enzymatic and chemical digestion, displaying a histological structure similar to that of terrestrial mammals [1]. Finally, the pyloric stomach is the last chamber and presents a mucosa containing specialized pyloric glands involved in chemical and enzymatic digestion [6]. At the beginning of the intestine, a dilation of the gut, namely the duodenal ampulla, is present, where the hepatopancreatic duct empties in most species. Although it may be mistaken for a gastric compartment, it represents the most proximal portion of the intestine [1]. One of the most remarkable features in cetaceans of the *Delphinidae* and *Phocoenidae* families is the absence of macroscopic differentiation between the small and large intestine [7], with some authors referring to the entire gut as the small intestine [8]. To address this issue, the intestinal vascularization pattern of the bottlenose dolphin was studied to attempt the differentiation between intestinal compartments [9]. In addition, while mysticetes possess a caecum and a vermiform appendix, these structures appear to be absent in the odontocetes [4], with exceptions such as the caecum observed in a river dolphin (*Platanista gangetica*) [10]. The intestine continues towards the rectum and the anal canal, where the anal tonsil, a relevant lymphoid tissue, is located [11].

Gut-associated lymphoid tissue (GALT) comprises lymphoid structures in the mucosa and submucosa of the GIT, playing a central role in immune tolerance and pathogen defence [12]. GALT includes Peyer’s patches (organized aggregates mainly found in the ileum of most domestic mammals), diffuse lymphoid follicles along the intestine, and isolated immune cells such as intraepithelial lymphocytes [13]. In cetaceans, studies in belugas reported diffuse lymphocytes and follicles but no true Peyer’s patches [14]. In young individuals, the distal rectum contains prominent lymphoid structures that disappear just before the anal canal (“pigskin appearance”), which regress with age, remaining only scattered lymphocytes [11]. More recent studies have confirmed the presence of Peyer’s patches in juvenile cetaceans, especially in the mid- to distal gut [15], and highlight age-related involution of GALT in bottlenose dolphins [16].

In addition, GALT includes the anal tonsil, which refers to a macroscopically visible lymphoid structure located in the anal canal, found in some cetacean species [7,14]. The anal tonsil may function in antigen presentation, reacting to waterborne antigens entering during diving [11]. It consists of lymphoid cell clusters with epithelial crypts and occasional mucous glands. In advanced involution stages, glandular structures suggest a shift from immune to mechanical functions, such as lubrication [16].

In contrast to the GIT, the histological structure of the lymph nodes (LNs) has been described in several studies conducted on cetaceans [11,17], and a morphology similar to that of terrestrial mammals’ LNs has been observed. However, in the odontocetes harbour porpoise (*Phocoena phocoena*) and common dolphin (*Delphinus delphis*), it has been reported that a pig-like structure may occasionally be present [18], in which the cortical and medullary regions exhibit an inverted pattern.

Understanding the normal histological structure of the GIT and its associated lymphoid tissue in cetacean species is essential for assessing their health status. Such baseline information allows researchers to distinguish physiological, anatomical and immunological variations from pathological changes, which is particularly important given the limited accessibility to these specimens. Moreover, characterizing the GALT provides insight into gut immunity, a field that remains largely unexplored in cetaceans, and establishes a foundation for future studies supporting conservation efforts.

Taking into account that lack of information, the aims of this study were to (i) histologically characterize the GIT and associated lymphoid tissue of a harbour porpoise that died due to *bycatch*; (ii) quantify and describe the distribution of macrophages, T lymphocytes (TL) and B lymphocytes (BL) in the lymphoid tissue present in the GIT and regional LNs; and (iii) establish a reliable method for further histological structure studies of GIT and LNs in cetaceans.

## 2. Materials and Methods

### 2.1. Sampling

**Figure 1:**
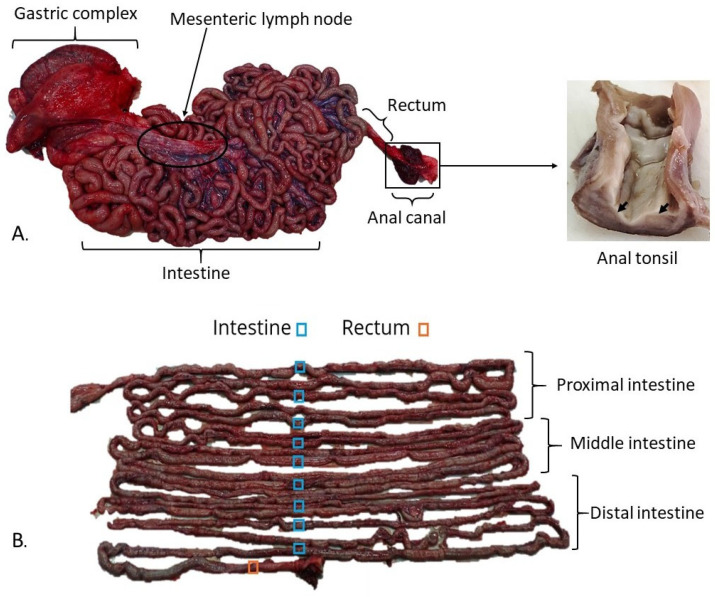
Macroscopical structure of the gastrointestinal tract of harbour porpoise (*Phocoena phocoena*). (**A**) Gastrointestinal segments are shown. The arrows point to the anal tonsil. (**B**) The nine intestinal samples collected are highlighted.

One subadult male harbour porpoise that died because of *bycatch* in the Bay of Biscay (Cantabrian Sea) was used in this study. Necropsy was performed in 48 h, and samples from the GIT and associated lymphoid tissue were collected and fixed in 10% buffered formalin for further histological and immunohistochemical (IHC) studies. GIT samples included all three chambers from the gastric complex (i.e., forestomach, main stomach and pyloric stomach), the transition between the gastric compartment and intestine, duodenal ampulla, intestine segments and rectum (Figure 1A). The intestine (18.5 m) was divided into three regions: proximal, middle and distal, with a length of approximately six metres each. Three samples were taken from equidistant points from each region, making a total of nine segments from the intestine (Figure 1B). Intestinal samples were collected in a closed state to maintain the tubular shape, and formalin was carefully injected into the lumen to attempt to preserve the mucosal integrity. LNs were taken from the gastrosplenic and pancreatic regions. Additionally, the mesenteric LN (subdivided into proximal, middle, and distal portions) and the rectal LN were sampled. Since the anal canal from this animal was unavailable, it was collected with its associated anal tonsil from a subadult female porpoise bycaught in the same area to complete the study (Figure 1A). Pathological evaluation confirmed the absence of any disease.

### 2.2. Histology and Immunohistochemistry

For each sample (n = 16), serial paraffin-embedded sections (3 µm) were used for histological and IHC studies. The staining techniques included the hematoxylin and eosin standard stain and Masson’s trichrome stain for specific visualization of the connective tissue.

IHC consisted of the detection of three cell populations using different primary antibodies: ionized calcium-binding adaptor molecule 1 (IBA1) for macrophages, CD3 for TL and CD20 for BL. After being deparaffinized, antigen retrieval was carried out with sodium citrate buffer (10 mmol/L, pH 6.0) with heat induction by microwave for 20 min. Endogenous peroxidase activity was subsequently blocked by incubation in hydrogen peroxide (0.5%) solution in distilled water for 30 min at room temperature. The tissue sections were then incubated overnight at 4 °C in a humidified chamber with commercial monoclonal or polyclonal antibodies diluted in Tris-buffered saline with bovine serum albumin (TBS + BSA) 0.1% (Table 1), washed with TBS 1x, and incubated with a secondary antibody (Vector Laboratories, Newark, CA, USA), diluted 1:200 in TBS + BSA 0.1% (Table 1), followed by incubation with the avidin–biotin–peroxidase complex reagent method (ABC Standard, PK-4000, Vector Laboratories, CA, USA) in TBS 1× for 30 min. Labelling was visualized using the Vector^®^ NovaRed™ peroxidase substrate kit (SK-4800, Vector Laboratories, CA, USA) as chromogen substrate. Slides were counterstained with Mayer’s haematoxylin, dehydrated and mounted with DPX (06522, Fluka, Sigma, St. Louis, MO, USA). The negative control consisted of an additional slide without the primary antibody. Lymph node tissue from a road-killed badger was used as a positive control for the three antibodies (Supplementary Materials, Figure S1).

### 2.3. Measurement of Histological Layer Thickness in the GIT

**Figure 2:**
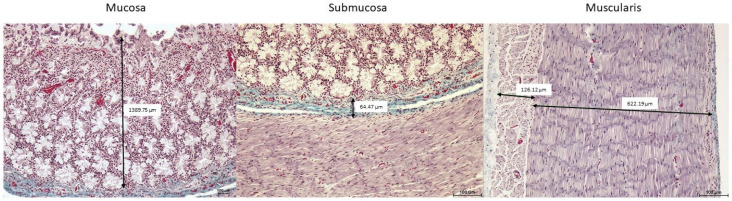
Microphotographs of the middle intestine showing how measurements of the different tissue layers were taken. Masson’s trichrome staining. 100× magnification.

Samples were examined using a Nikon E600 microscope and a Nikon DS-FI1 camera. Image analysis v. 6.10.02 software (Nikon NIS-Elements Br, Nikon, Tokyo, Japan) was used to measure the thickness of the different layers of the GIT (Figure 2). Five measurements were taken for each sample: mucosa or mucosa–submucosa layers (when muscularis mucosae was absent), submucosa, and internal and external muscularis layers. A total of 350 measurements were taken.

### 2.4. Evaluation and Quantification of Cell Types in the Gastrointestinal Tract (GIT) and Lymph Nodes (LNs)

Immunostained tissue sections were scanned at the Microscopy Service of the University of León. An Olympus BX51 microscope (Olympus, Tokyo, Japan) and an Olympus XC10 camera (Olympus, Tokyo, Japan) were used. The digitally scanned samples were then analyzed using the QuPath v. 0.5.1 image analysis software [19] (QuPath, University of Edinburgh, Scotland).

For quantification of cells in the GIT, five fields were randomly sampled from the mucosa, submucosa, and muscularis layers (a total of 15 counts/sample). Regarding LNs, five fields were randomly sampled from the diffuse lymphoreticular tissue of the cortex, lymphoid follicles, and medulla, obtaining 15 cell counts/LN.

For cell counting in each field, an area of 62,000 μm^2^ was delimited, yielding the following results: total number of cells detected, number of positive and negative cells, percentage of positive cells detected in the area, and number of positive cells per mm^2^. A total of 745 positive cell count fields were obtained.

### 2.5. Statistical Analysis

Statistical analysis was performed using IBM SPSS Statistics v. 22.0 software (IBM SPSS Statistics, IBM, Armonk, NY, USA). Since the values did not meet the assumptions of normality (Kolmogorov–Smirnov and Shapiro–Wilk tests), nonparametric tests were used. Statistical significance between categories was assessed using the Mann–Whitney U test and the Kruskal–Wallis test to compare two or more independent groups, respectively. When statistical differences were found, pairwise comparisons were conducted. The level of statistical significance was set at *p* = 0.05.

## 3. Results and Discussion

### 3.1. Histological Structure of the Gastrointestinal Tract (GIT)

**Figure 3:**
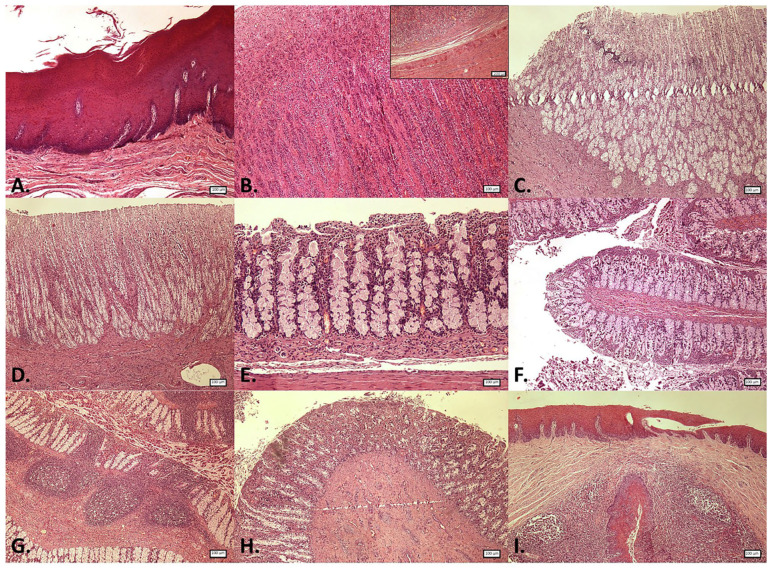
Microphotographs of the different segments of the gastrointestinal tract in harbour porpoise (*Phocoena phocoena*). Hematoxylin and eosin staining. (**A**) Forestomach. (**B**) Main stomach: mucosa. Inset: mucosa–submucosa. (**C**) Pyloric stomach. (**D**) Duodenal ampulla. (**E**) Proximal intestine. (**F**) Middle intestine. (**G**) Distal intestine. (**H**) Rectum. (**I**) Anal canal. Microphotographs (**A**–**D**,**F**–**I**) at 40× magnification; (**E**) and inset at 100× magnification.

**Figure 4:**
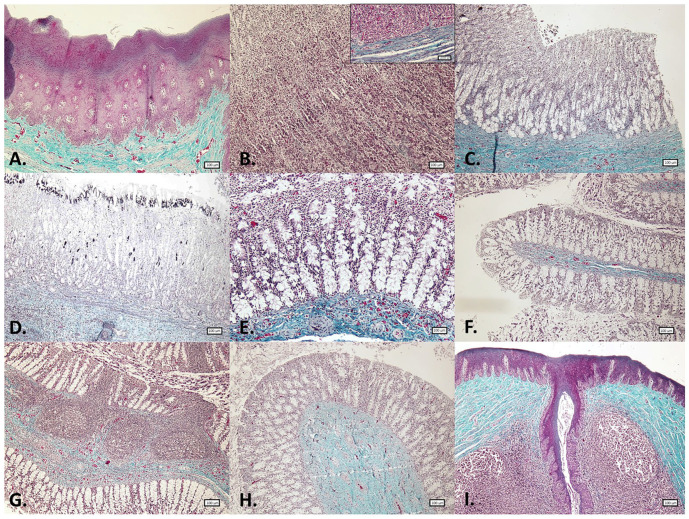
Microphotographs of the different segments of the gastrointestinal tract in harbour porpoise (*Phocoena phocoena*). Masson’s trichrome staining. (**A**) Forestomach. (**B**) Main stomach: mucosa. Inset: mucosa–submucosa. (**C**) Pyloric stomach. (**D**) Duodenal ampulla. (**E**) Proximal intestine. (**F**) Middle intestine. (**G**) Distal intestine. (**H**) Rectum. (**I**) Anal canal. Microphotographs (**A**–**D**,**F**–**I**) at 40× magnification; (**E**) and inset at 100× magnification.

Main histological findings in the GIT are shown in Table 2. In the histological study of the gastric complex, the adaptation of the forestomach to mechanical digestion [1] was reflected in the robust lining epithelium (Figure 3 and Figure 4), the absence of glands, and the marked development of the muscularis (Table 2). In turn, the pronounced development of the mucosa (Table 2) and the high number of secretory cells in the main stomach highlighted the relevance of this chamber in chemical and enzymatic digestion. Although the literature considers that the duodenal ampulla and the remaining segments of the duodenum share the same structure [1], in this study, the ampulla exhibited a greater development of the muscularis mucosae and lacked folds (Figure 3 and Figure 4), which were present in the subsequent intestinal sections. Furthermore, the thickness of all histological layers was notably greater in the duodenal ampulla compared to the other intestinal regions (see Table 2). Consistent with previous descriptions, folds were observed in the intestinal mucosa [1,4,20], being more abundant in the proximal sections and gradually decreasing towards the more distal regions of the intestine.

The presence of glands in the submucosa has been reported to vary among different cetacean species [4]. In this study, no glands were observed in any of the gastrointestinal segments examined, and submucosal plexus was present only in the gastric chambers and the rectum. Regarding villi, while some authors reported the absence of well-defined villi [8] or even their complete absence in the most distal regions of the intestine [21], others described short and thin villi [1,20]. In this porpoise, no villi were observed. Given these discrepancies, it is important to consider the challenges associated with cetacean sample collection and the rapid degeneration of intestinal mucosa after death. In the same line, no muscularis mucosae was observed in the rectum, which contrasts with its presence in other terrestrial mammalian species [22,23]. Also, numerous lymphoid follicles have been identified in the mucosa of this segment in other studies [5], although they were not observed in the harbour porpoise studied.

Finally, the statistical analysis of the thickness of the nine intestinal samples, taken from the duodenal ampulla to the rectum, showed no significant differences, making it impossible to distinguish between the small and large intestine based solely on wall thickness, unlike in other odontocete species, such as the franciscana (*Pontoporia blainvillei*), where it can be clearly differentiated [24].

### 3.2. Distribution of Immune Cells Within Gastrointestinal Tract (GIT) and Associated Lymphoid Tissue

In the GIT, the most frequently detected cells were macrophages, followed by BL and TL. Likewise, the submucosa represented the layer with the highest density of immunopositive cells, followed by the mucosa and muscularis.

**Figure 5:**
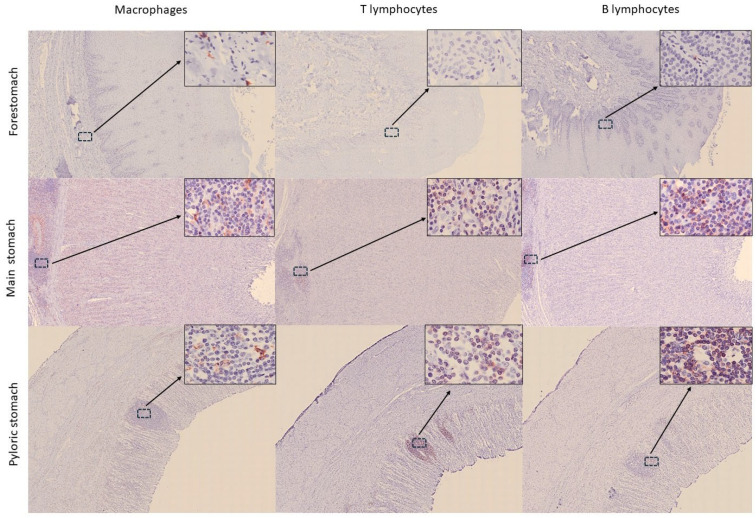
Distribution of immune cells (macrophages—IBA1, T lymphocytes—CD3, and B lymphocytes—CD20) in the three gastric chambers of harbour porpoise (*Phocoena phocoena*). Note that the presence of immune cells is lower in the forestomach, mainly T lymphocytes. Microphotographs at 25× magnification; insets at 400× magnification.

Within the gastric chambers, the pyloric stomach exhibited the highest percentage of immunopositive cells, with an average of 6.73% of IBA1-stained cells in the mucosa, 8.83% in the submucosa, and 3.44% in the muscularis layer. However, the higher cellular positivity in this chamber, at its transition with the intestine and in the duodenal ampulla itself, is likely associated with the presence of lymphoid cell clusters (Figure 5).

**Figure 6:**
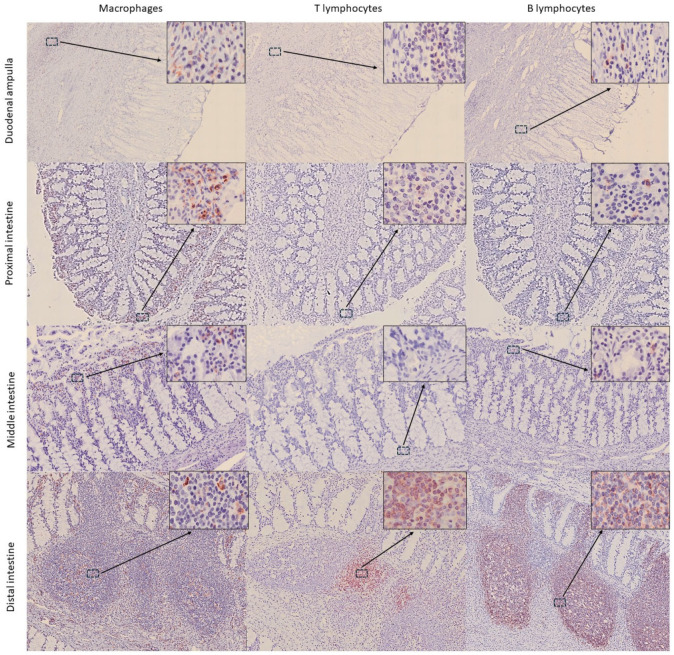
Distribution of immune cells (macrophages—IBA1, T lymphocytes—CD3, and B lymphocytes—CD20) in the different intestinal segments of harbour porpoise (*Phocoena phocoena*). Aggregates of immune cells can be observed in the distal intestine. Microphotographs at 25× magnification; insets at 400× magnification.

**Figure 7:**
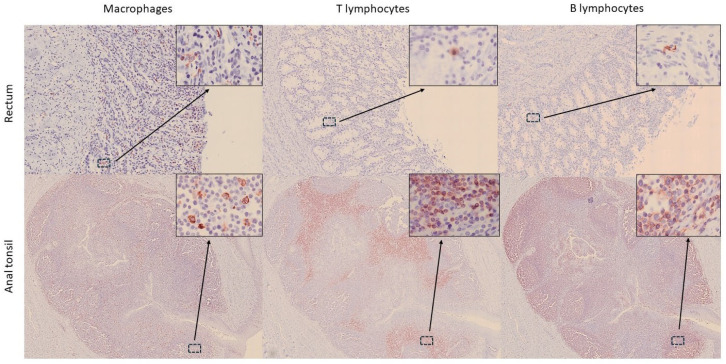
Distribution of immune cells (macrophages—IBA1, T lymphocytes—CD3, and B lymphocytes—CD20) in the rectum and anal tonsil of harbour porpoise (*Phocoena phocoena*). Big clusters of immune cells are observed in the anal canal. Microphotographs at 25× magnification; insets at 400× magnification.

**Figure 8:**
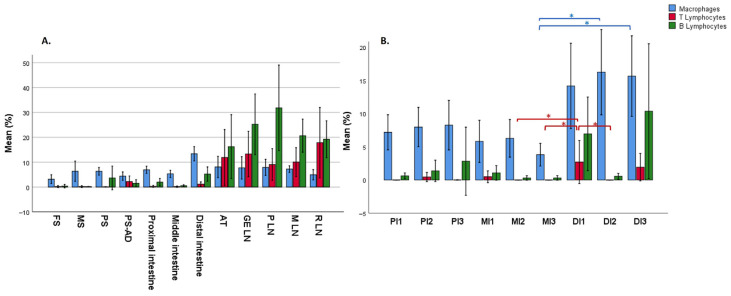
Percentage of immune cells along the gastrointestinal tract and lymph nodes in harbour porpoise (*Phocoena phocoena*). (**A**) Mean percentage of immune cells (macrophages—IBA1, B lymphocytes—CD20, and T lymphocytes—CD3). FS, forestomach; MS, main stomach; PS, pyloric stomach; DA, duodenal ampulla; AT, anal tonsil; GE LN, gastrosplenic lymph node; P LN, pancreatic lymph node; M LN, mesenteric lymph node; R LN, rectal lymph node. (**B**) Samples collected from cranial to distal segments of proximal intestine (PI1-PI2-PI3), middle intestine (MI1-MI2-MI3), and distal intestine (DI1-DI2-DI3). When significant differences were found, pairwise comparisons were conducted (*). Error bars represent 95% confidence intervals.

In the intestine, significant differences were observed in the distribution of the three cell populations across the nine collected segments (*p* = 0.005, *p* = 0.000, and *p* = 0.034 for macrophages, TL, and BL, respectively). Macrophages were mainly located in the apical region of the mucosa, forming an almost continuous layer of cells (Figure 6). TL and BL were diffusely distributed near the lamina propria of the mucosa layer and predominated in the lymphoid aggregates observed in the submucosa, with BL being the most abundant cells (Figure 6). While immune cells in the proximal and middle intestine samples appeared only occasionally as aggregates, in the distal segment, aggregates were more frequently observed, forming well-defined lymphoid follicles in the submucosa and infiltrating the mucosa layer (Figure 6). This was reflected in the marked increase in immune cell detection in this last segment (Figure 7 and Figure 8). Furthermore, the follicles found in the distal portions were structures similar to the Peyer’s patches of terrestrial mammals [23]. These structures appeared as well-defined, circular lymphoid follicles in the submucosa layer, either isolated or in contact with nearby follicles, sometimes infiltrating the entire thickness of the mucosa (Figure 6). They were frequently detected in mucosal folds, and no well-defined germinal centres were observed. Also, macrophages were diffusely distributed within these Peyer’s patches, BL were the most abundant cells predominating in all follicles and mucosal infiltrations, and TL were diffusely distributed, mainly in the interfollicular areas. Although these lymphoid structures are consistent with those described by Silva et al. (2016) [15], the authors reported their presence in the middle region and final third of the intestine, whereas in this study, they were exclusively observed in the final third. In this line, Cowan and Smith (1999) [11] described the presence of a continuous layer of lymphoid structures in the lamina propria of the distal intestinal segments beyond the splenic flexure, without providing a detailed description of the tissue morphology. Moreover, the authors suggested that this segment was analogous to the vermiform appendix. In comparison to other terrestrial mammals, where Peyer’s patches are most frequently found in the ileum [22,25], our findings suggest that, in the harbour porpoise, these lymphoid structures are located further distally in the intestine, where the colon would typically be expected. However, it should be considered that the porpoise studied in this work was a subadult, in which Peyer’s patches were likely undergoing involution [16].

In addition, immune cells were identified in the anal canal, diffusely distributed within the keratinized stratified epithelium and the mucosa–submucosa (Figure 3 and Figure 4). However, most immune cells were concentrated in large aggregates of lymphoid tissue surrounding the epithelial crypts, corresponding to the anal tonsil (Figure 7). Although the histological structure observed was consistent with previous descriptions [11,15], the use of IHC techniques in the present study provided deeper insights into the cellular composition of this organ. In this regard, while macrophages were the most frequently detected immune cells throughout the GIT, in the anal tonsil, they were less abundant than TL and BL, with the latter being the most prevalent (average percentage of positive cells in the mucosa–submucosa: 10.7%, 17.8%, and 24.3%, respectively) (Figure 8). The higher proportion of BL may be related to a stronger local humoral immune response at this level [12]. Regarding their distribution, BL and macrophages were observed diffusely in the epithelium and lamina propria–submucosa, as well as within the lymphoid aggregates, whereas TL were detected exclusively between these clusters. Those aggregates showed well-defined follicles, with macrophages diffusely distributed, TL predominating in the interfollicular regions, and BL distributed both diffusely and associated with the mantle and germinal centre of the lymphoid follicles. Interestingly, the cellular detection levels of macrophages, TL, and BL were comparable to those observed in the LNs analyzed in this study, where no significant differences were found. Therefore, based on its cellular composition, the anal tonsil might be considered a lymphoid organ analogous to the LNs.

**Figure 9:**
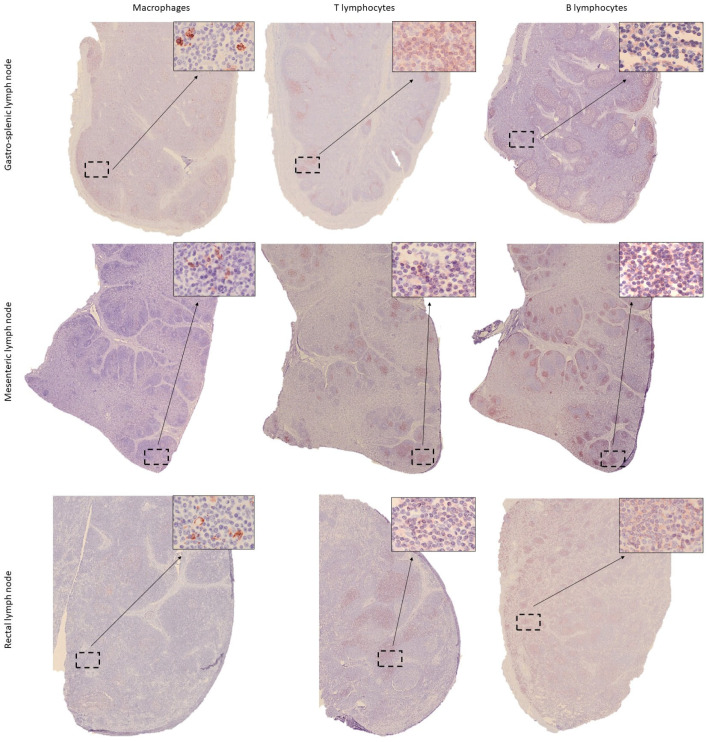
Distribution of immune cells (macrophages—IBA1, T lymphocytes—CD3, and B lymphocytes—CD20) in the gastrosplenic, proximal mesenteric and rectal lymph nodes of harbour porpoise (*Phocoena phocoena*). Microphotographs at 25× magnification; insets at 400× magnification.

Although some publications have described the LNs of the harbour porpoise as having an inverted structure similar to that of pigs [18], in the present study, they exhibited a typical structure, with a well-differentiated cortex containing lymphoid follicles and a medullary region (Figure 9), consistent with descriptions in other cetacean species [20,26]. Overall, BL were the most frequently detected cells, followed by lower proportions of TL and macrophages (Figure 8). As expected, the cortical lymphoid follicles represented the region with the highest density of immunopositive cells, compared to the diffuse lymphoreticular tissue of both cortical and medullary regions (Figure 9). The percentage of macrophages did not show significant differences between the different LNs. In contrast, BL showed higher positivity in the cranial LNs, with the main detection in the pancreatic LN, followed by a gradual decrease in caudal LNs (Figure 8). In contrast, TL displayed an opposite pattern to that of BL, increasing in the caudal regions and reaching maximum positivity in the rectal LN (Figure 8).

A limitation of this work is the study of a single specimen; however, the inherent challenges in obtaining cetacean samples, particularly from healthy individuals, must be considered. In this line, our study provides preliminary baseline data on the normal histology of the GIT and associated lymphoid tissue in a porpoise. This information is relevant for detecting pathological changes in stranded or *bycatch* individuals and for understanding how the immune system contributes to gastrointestinal health. Given the inaccessibility of these animals, detailed knowledge of normal tissue structure and immune organization is particularly valuable. Furthermore, the GALT plays a central role in gut immunity, yet remains poorly understood in cetaceans. IHC resulted in a useful tool to detect immune cells in GALT, thus opening up the possibility of future immune response studies in cetaceans. By establishing these preliminary data, our work may contribute to highlighting gaps in knowledge as a framework for future research to support the conservation of these valuable animals.

## 4. Conclusions

This study provides the first detailed histological and immunohistochemical characterization of the GIT and associated lymphoid tissue in harbour porpoise. The results highlight structural adaptations in the GIT for digestion and regional variation in immune cell distribution, which enables microscopic identification of the distal intestinal segment, even though distinguishing between the small and large intestines remains unfeasible. Lymphoid follicles resembling Peyer’s patches were restricted to the distal intestine, and the anal tonsil showed features comparable to LNs, supporting its relevant role as a major lymphoid organ. LNs showed typical mammalian organization, with BL predominating and displaying craniocaudal variation opposite to that of TL. This study establishes preliminary information for future research on cetacean health.

## Figures and Tables

**Table 1:** Primary and secondary antibodies used for cellular type characterization.

Primary Antibody (Dilution)	Cell Type Detected	Clone No.	Source	Secondary Antibody (Dilution)	Catalogue No.	Source
IBA1 ^1^ (1:1000)	Macrophages	Polyclonal 019-19741	FLUJIFILM-Wako Chemicals Europe GmbH, Neuss, Germany	Goat anti-rabbit (1:200)	BA-1000-1.5	Vector Laboratories, Newark, CA, USA
CD3 (1:500)	T lymphocytes	Monoclonal NCL-L-CD3-565	Novacastra, Leica Biosystem, Newcastle, UK	Horse anti-mouse (1:200)	BA-2000-1.5	Vector Laboratories, Newark, CA, USA
CD20 (1:400)	B lymphocytes	Polyclonal PA5-16701	ThermoFisher, Waltham, MA, USA	Goat anti-rabbit (1:200)	BA-1000-1.5	Vector Laboratories, Newark, CA, USA

^1^ Ionized calcium-binding adapter molecule 1.

**Table 2:** Main histological findings in the gastrointestinal tract of harbour porpoise (*Phocoena phocoena*).

Structure	Mucosa	Submucosa	Muscularis
Forestomach (FS)	Stratified keratinized epithelium. Muscularis mucosae highly developed. Absence of glands. Thickness: 937 microns (µm).	No glands. Submucosal plexus. Thickness: 252 µm.	Layers difficult to differentiate. Myenteric plexus. Thickness: 1430 µm.
Main stomach (MS)	Simple columnar epithelium. Gastric pits with secretory cells. Muscularis mucosae less developed than in FS. Thickness: 3180 µm.	No glands. Submucosal plexus. Thickness: 403 µm.	Inner and outer layers easily distinguishable. Myenteric plexus. Thickness: 913 µm (inner) and 432 µm (outer).
Pyloric stomach (PS)	Simple columnar epithelium. Pyloric glands. Muscularis mucosae. Thickness: 1260 µm.	No glands. Submucosal plexus. Thickness: 292 µm.	Inner and outer layers easily distinguishable. Myenteric plexus. Thickness: 774 µm (inner) and 212 µm (outer).
Duodenal ampulla (DA)	Simple columnar epithelium. Mucous glands. Muscularis mucosae poorly developed. No folds. Thickness: 1140 µm.	No glands. No submucosal plexus. Thickness: 639 µm.	Inner and outer layers easily distinguishable. Myenteric plexus. Thickness: 712 µm (inner) and 512 µm (outer).
Proximal intestine 1 (PI1)	Simple columnar epithelium. Mucous glands. Muscularis mucosae very poorly developed. No villi. Numerous folds. Thickness: 272 µm.	No glands. No submucosal plexus. Thickness: 135 µm.	Inner and outer layers easily distinguishable. Myenteric plexus. Thickness: 245 µm (inner) and 61.8 µm (outer).
Proximal intestine 2 (PI2)	Simple columnar epithelium. Mucous glands. Muscularis mucosae very poorly developed. No villi. Numerous folds. Thickness: 461 µm.	No glands. No submucosal plexus. Thickness: 97.6 µm.	Inner and outer layers easily distinguishable. Myenteric plexus. Thickness: 629 µm (inner) and 162 µm (outer).
Proximal intestine 3 (PI3)	Simple columnar epithelium. Mucous glands. Muscularis mucosae very poorly developed. No villi. Numerous folds. Thickness: 361 µm.	No glands. No submucosal plexus. Thickness: 96.8 µm.	Inner and outer layers easily distinguishable. Myenteric plexus. Thickness: 629 µm (inner) and 191 µm (outer).
Middle intestine 1 (MI1)	Simple columnar epithelium. Mucous glands. Muscularis mucosae very poorly developed. No villi. Numerous folds. Thickness: 351 µm.	No glands. No submucosal plexus. Thickness: 63.9 µm.	Inner and outer layers easily distinguishable. Myenteric plexus. Thickness: 522.464 µm (inner) and 130 µm (outer).
Middle intestine 2 (MI2)	Simple columnar epithelium. Mucous glands. Muscularis mucosae very poorly developed. No villi. Few folds. Thickness: 423 µm.	No glands. No submucosal plexus. Thickness: 96.3 µm.	Inner and outer layers easily distinguishable. Myenteric plexus. Thickness: 456 µm (inner) and 91.4 µm (outer).
Middle intestine 3 (MI3)	Simple columnar epithelium. Mucous glands. Muscularis mucosae very poorly developed. No villi. Few folds. Thickness: 373 µm.	No glands. No submucosal plexus. Thickness: 111 µm.	Inner and outer layers easily distinguishable. Myenteric plexus. Thickness: 408 µm (inner) and 124 µm (outer).
Distal intestine 1 (DI1)	Simple columnar epithelium. Mucous glands. Muscularis mucosae very poorly developed. No villi. Few folds. Thickness: 328 µm.	No glands. No submucosal plexus. Thickness: 142 µm.	Inner and outer layers easily distinguishable. Myenteric plexus. Thickness: 359 µm (inner) and 112 µm (outer).
Distal intestine 2 (DI2)	Simple columnar epithelium. Mucous glands. Muscularis mucosae very poorly developed. No villi. Few folds. Thickness: 364 µm.	No glands. No submucosal plexus. Thickness: 142 µm.	Inner and outer layers easily distinguishable. Myenteric plexus. Thickness: 373 µm (inner) and 90.3 µm (outer).
Distal intestine 3 (DI3)	Simple columnar epithelium. Mucous glands. Muscularis mucosae very poorly developed. No villi. Few folds. Thickness: 344 µm.	No glands. No submucosal plexus. Thickness: 143 µm.	Inner and outer layers easily distinguishable. Myenteric plexus. Thickness: 518 µm (inner) and 103 µm (outer).
Rectum (R)	Simple columnar epithelium. Mucous glands. No muscularis mucosae. Wide folds. Thickness: 737 µm.	No glands. Submucosal plexus present.	Inner and outer layers easily distinguishable. Myenteric plexus. Thickness: 808 µm (inner) and 353 µm (outer).
Anal canal (AC)	Stratified keratinized epithelium. Crypts with stratified keratinized epithelium. No glands. No muscularis mucosae. Thickness: 3400 µm.	No glands. No submucosal plexus. Large lymphoid aggregates (anal tonsil).	Inner and outer layers easily distinguishable. Myenteric plexus. Thickness: 854 µm (inner) and 544 µm (outer).

## Data Availability

All data can be found in the manuscript.

## Supplementary Materials

The following supporting information can be downloaded at https://www.mdpi.com/article/10.3390/ani15223277/s1. Figure S1. Microphotographs of a badger’s lymph node used as positive and negative controls in the immunohistochemical technique (macrophages—IBA1, T lymphocytes—CD3, and B lymphocytes—CD20). 400× magnification.

## Abbreviations

The following abbreviations are used in this manuscript:

BL	B lymphocytes
TL	T lymphocytes
GALT	Gut-associated lymphoid tissue
GIT	Gastrointestinal tract
IBA1	Ionized calcium-binding molecule 1
IHC	Immunohistochemistry
LN	Lymph node
