# Supplementary figures and images for: First Histological Study of the Gastrointestinal Tract and Associated Lymphoid Structures of a Harbour Porpoise (Phocoena phocoena)
Source: Animals (Basel). 2025 Nov 13;15(22):3277. doi: 10.3390/ani15223277 (PMC12649499; doi:10.3390/ani15223277)

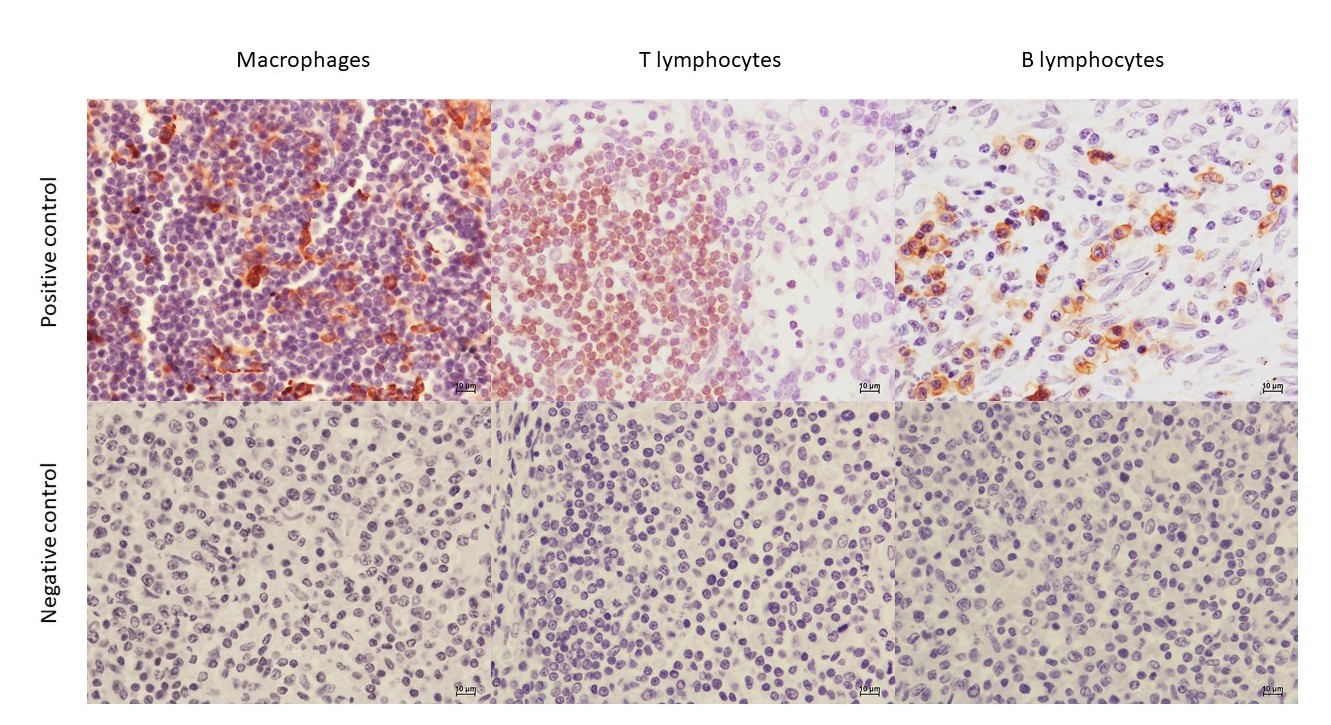

Supplement: Supplementary file 1 [file animals-15-03277-s001.zip › animals-3929708-supplementary.jpg]
